# Sudden Death and Coronary Artery Anomalies

**DOI:** 10.3389/fcvm.2021.636589

**Published:** 2021-03-18

**Authors:** Stefania Rizzo, Monica De Gaspari, Carla Frescura, Massimo Padalino, Gaetano Thiene, Cristina Basso

**Affiliations:** ^1^Cardiovascular Pathology, Azienda Ospedaliera, Department of Cardiac, Thoracic and Vascular Sciences, and Public Health, University of Padua, Padua, Italy; ^2^Pediatric and Congenital Cardiac Surgery, Department of Cardiac, Thoracic and Vascular Sciences, and Public Health, University of Padua, Padua, Italy

**Keywords:** coronary anomalies, pathology, sudden death, diagnosis, surgical procedures

## Abstract

Congenital coronary artery anomalies (CAA) include a wide spectrum of malformations present at birth with various clinical manifestations and degrees of severity. Patients may be asymptomatic, and CAA may be an incidental finding during cardiac imaging or at autopsy. However, in other cases, ischemia-related signs and symptoms, leading to an increased risk of sudden cardiac death (SCD), often as first presentation may occur. In this chapter, we discuss the normal anatomy of the coronary arteries (CA) and the pathology of CAA at risk of SCD, including our experience with victims of SCD among the young population (age <40 years) and among athletes.

## Normal Anatomy of the Coronary Arteries (CA)

Normally, two main CA, the right (RCA) and the left main (LCA), the latter branching into the left circumflex artery (LCX) and the left anterior descending artery (LAD), arise from the aortic right anterior and left anterior sinuses of Valsalva, respectively, close to the sino-tubular junction, without any relation to the pulmonary trunk. Variations on normal anatomy are the separate origin of the conal artery and RCA from the right coronary sinus as well as that of the LCX and the LAD from the left coronary sinus. A coronary ostium may originate from a higher position, up to 2.5 mm at maximum, compared to the normal site at the sino-tubular junction (1). Coronary dominance (right, left, co-dominant circulation) is also considered a variation of the normal. The main CA normally run in the subepicardium of the atrioventricular and interventricular grooves, dividing into branches which supply the atria and the ventricles.

## Coronary Artery Anomaly (CAA)

CAA is a rare disorder, reported in <1% of the general population on the basis of coronary imaging techniques and autopsy (2, 3). Although rare, CAA might precipitate myocardial ischemia at risk of sudden cardiac death (SCD), even in the young and in athletes.

Several classifications have been proposed for CAA (3, 4). The classification by Angelini (4) is based on anatomical features, and three categories are recognized: anomalies of origin and course; anomalies of intrinsic CA anatomy; and anomalies of coronary termination.

While anomalies of origin and course will be discussed in depth because of their potential link to SCD, anomalies of intrinsic CA anatomy and termination will be briefly commented on. The latter includes ostial stenosis/atresia and hypoplasia. CA ostial stenosis/atresia is an extremely rare anomaly leading to collateral vessels formation from the normal CA, usually inadequate for satisfying myocardial oxygen demand. The clinical presentation is usually in the first year of life. Hypoplastic CA refers to a narrowed lumen (<1.5 mm) of one or two of the three main epicardial CA (5). However, caution should be used not to confound extreme right or left dominant patterns with CA hypoplasia.

Anomalies of coronary termination correspond to coronary fistulae, characterized by a connection between the CA and a cardiac chamber or intrathoracic great vessel, leading to left-to-right shunts and myocardial ischemia. Moreover, termination in a low-pressure space causes enlargement and tortuosity of the fistulous CA at risk of aneurysmatic dilatation and rupture (6).

While in the past these CAA could only be described at autopsy, currently they can be effectively detected with non-invasive diagnostic imaging, thanks to enormous technological advancements.

The most practical non-invasive diagnostic tool is transthoracic two-dimensional echocardiography, which in experienced hands can identify these anomalous origins *in vivo* with a good sensivity, although it remains more effective and easier in the pediatric population. Transesophageal echocardiography is much more sensitive but is a semi-invasive tool. However, nowadays, axial computed tomography (CT) and/or magnetic resonance (MR) imaging are reliable non-invasive tools for diagnosing CAA and are proposed and accepted worldwide as the gold standard for the identification of an anomalous origin and course. Last, but not least, cardiac stress test and myocardial scintigraphy are complementary investigations that may help in assessing the functional status, and guide surgical indication.

## CAA and SCD: Risk IS Not the Same For All

According to the autopsy guidelines for the study of SCD of the Association for European Cardiovascular Pathology (7), the degrees of certainty (i.e., certain, highly probable, or uncertain) in defining the causative role of various CAA in SCD are different, along with the recommendations for management and sport eligibility. Only the origin from the pulmonary trunk is considered as a certain cause, with the origin of LCA from the opposite right sinus of Valsalva considered as a highly probable cause and the remaining (RCA from left, LCX from right and retroaortic course, high take off and myocardial bridge) considered as uncertain causes of SCD.

### Anomalous Origin of CA From the Opposite Aortic Sinus

**Figure 1:**
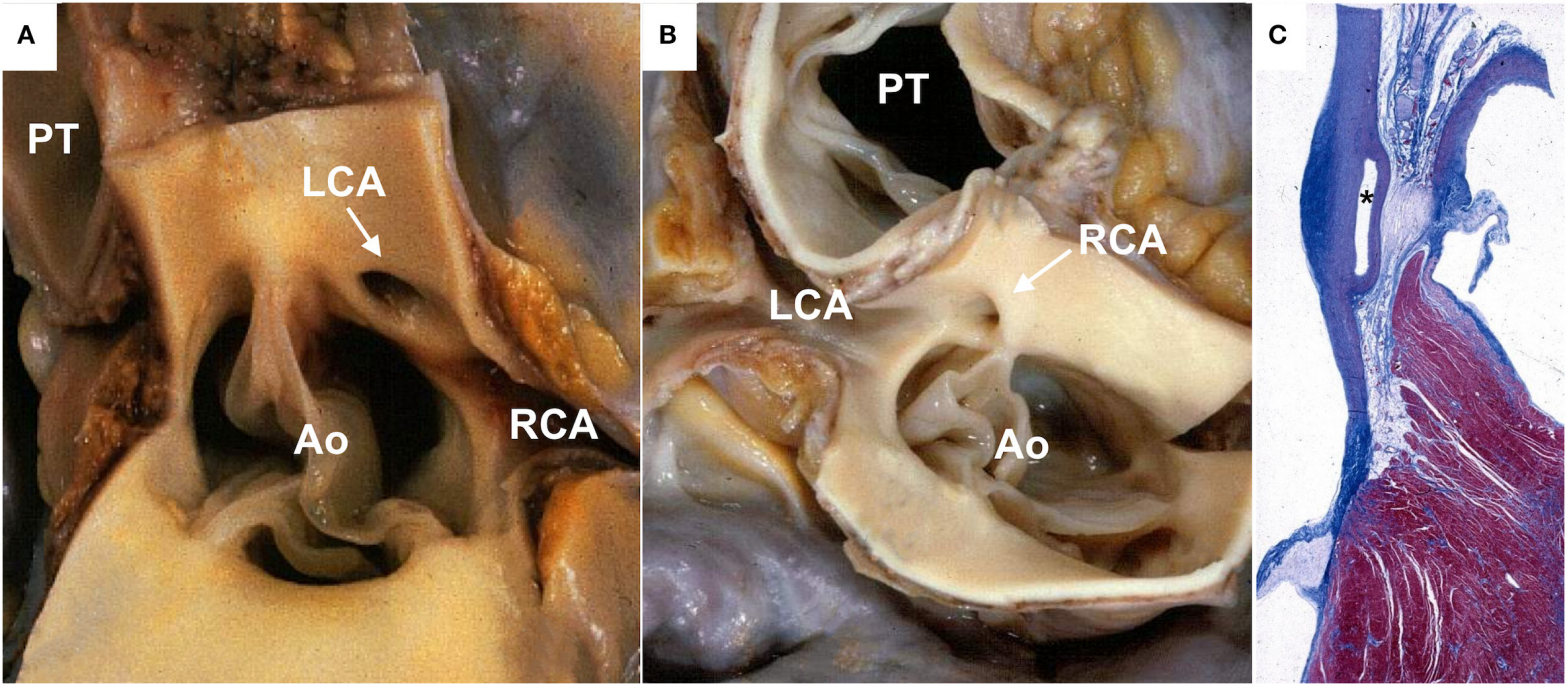
Anomalous origin of a coronary artery from the contralateral aortic sinus in sudden cardiac death cases. **(A)** Gross view of heart specimen showing the left coronary artery arising from the right aortic sinus close to the right coronary ostium (arrows) with a slit-like lumen. **(B)** Gross view of heart specimen showing the right coronary artery arising from the left aortic sinus, close to the left coronary ostium (arrow). **(C)** Histologic section showing the interarterial course of the left coronary artery between the aorta and the pulmonary trunk (asterisk). Ao, aorta; LCA, left coronary artery; PT, pulmonary trunk; RCA, right coronary artery.

The anomalous origin of a CA from the contralateral sinus of Valsalva (also known as anomalous aortic origin of CA) is the most common life-threatening anomaly associated with an increased risk of SCD, especially when the CAA has a proximal intramural and interarterial course between the aorta and the pulmonary artery (8–11). According to the proximal course of the anomalous CA, there are four subtypes: anterior to the pulmonary trunk (pre-pulmonic), posterior to the aorta (retroaortic), septal (sub-pulmonic), or between the pulmonary artery and the aorta (interarterial). The latter has been associated with an increased risk of SCD, especially in young athletes. Several explanations have been proposed: a slit-like lumen of the anomalous CA (11), an associated intramural course of the anomalous CA within the aortic wall, and a compression between the aortic root and the pulmonary trunk under effort, resulting in ischemia (11, 12). Barth and Roberts reported that in 38 autopsy patients with an LCA arising from the right aortic sinus with an interarterial course, 29 died suddenly in the first two decades of life and 28 during exercise (13). The origin of the LCA from the right sinus is considered more malignant, probably because of the wider myocardial territory at risk of ischemia. However, both RCA and LCA origins from the contralateral sinus increase the risk of SCD (Figure 1) (14). SCD may be the first manifestation of the disease, although patients may present with symptoms like syncope or chest pain (15–17). Moreover, myocardial necrosis and replacement-type fibrosis can trigger life-threatening ventricular arrhythmias. The anomalous origin of the LCA from the posterior aortic sinus is quite rare but may be associated with SCD as well.

The anomalous origin of the LCX from the right sinus of Valsalva or from the RCA is the second most common CAA (15), usually considered a benign condition since the course of the ectopic LCX is retroaortic. However, under effort, lumen stenosis due to compression by the dilated aortic root may occur. Cases of ischemia-related cardiovascular events, and less commonly unexpected arrhythmic SCD, have been described (7, 11).

### Anomalous Origin of CA From the Pulmonary Artery

An anomalous origin of the LCA from the pulmonary artery (ALCAPA), also called Bland-White-Garland syndrome (18), is a rare but potentially life-threatening CAA, characterized by a reverse flow into the pulmonary artery. An anomalous origin of the RCA from the pulmonary artery, or ARCAPA, is less frequent (19, 20). Most patients, if untreated and undiagnosed, develop myocardial ischemia and heart failure in infancy, and usually die within the first year of life. In fact, as pulmonary vascular resistances decrease physiologically, there is a reduction of the flow in the LCA. However, occasionally some patients may remain asymptomatic and survive into adulthood (21, 22). Depending on collateral vessels growth, we recognize two types of ALCAPA: the “adult type” with well-developed collaterals (23) and the “infant type” with poor collaterals and early onset of symptoms when pulmonary arterial pressure decreases. Although SCD may occur (24), the usual and most common clinical manifestation of ALCAPA is congestive heart failure.

### Single CA

This is a very rare condition in which only one CA arises from the aorta. A single CA may originate either from the left or the right Valsalva sinus and may coexist with other congenital anomalies. The single CA may take the course of either an RCA or an LCA and divide shortly from its origin into two or three of the main coronary branches. Lipton et al. (25) proposed an anatomical classification of single CA based on the location of the ostium, anatomical distribution, and course. Although single CA may be compatible with a normal life expectancy, thanks to the development of collateral branches, patients are at increased risk of myocardial ischemia and SCD when a major CA branch courses between the pulmonary artery and the aorta (26), especially when the single CA originates from the right sinus.

## A Still Controversial Risk for SCD: High Take-Off CA and Myocardial Bridge

### High Take-Off of a CA

The location of a CA ostium above the limit of 2.5 mm (1) has been observed in unexplained SCD, especially when the ostium is funnel-like with a narrowed lumen, and the course is intra-aortic before reaching the aortic root and then the atrioventricular (AV) sulcus (27–29). Intramural aortic course and compression during aortic dilatation under effort may account for lumen stenosis and inadequate myocardial supply. However, the clinical significance of this anomaly remains controversial (7).

### Myocardial Bridge

**Figure 2:**
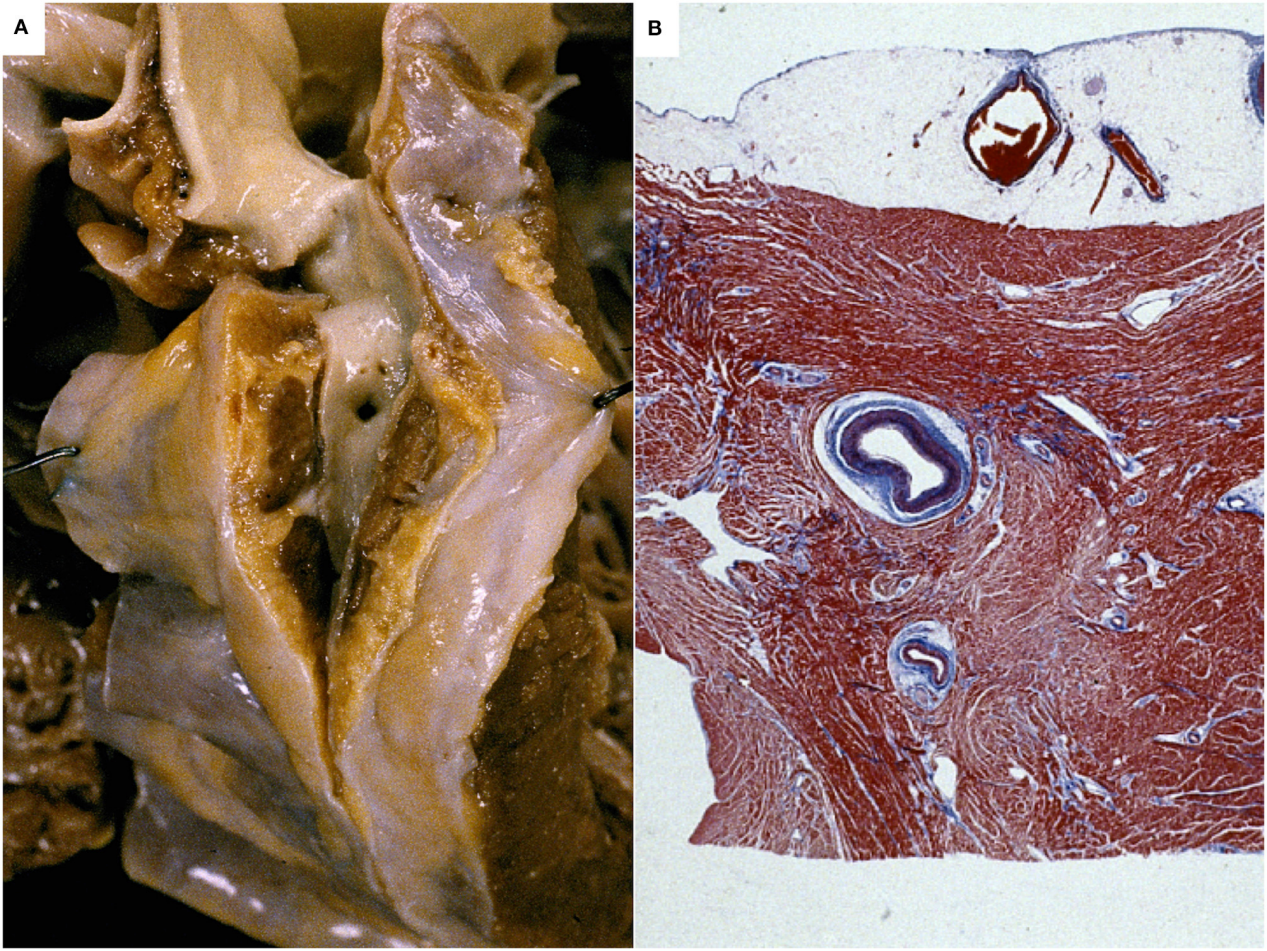
Myocardial bridge of the left coronary artery in an sudden cardiac death case. A segment of the left coronary artery runs deep in the myocardium. Gross view of the heart **(A)** and histology (Heidenhain stain) **(B)**.

This condition is a pure anomaly of the coronary course, while the origin and ostial features are usually perfectly normal. There are some doubts whether myocardial bridge constitute an anomaly or a normal variant, according to its frequency in the general population in imaging or autopsy studies (30). A myocardial bridge is defined as an atypical course of a CA intramyocardially, usually the proximal and mid-segment of the LAD, which may result in compression of the vessel during systole (milking effect) (Figure 2). Myocardial bridge may lead to ischemia, when characterized by a deeper (5 mm) and longer (2–3 cm) intramyocardial course, with the myocardium encircling the intramural segment acting like a sphincter (31, 32).

Myocardial bridge is found frequently in patients with hypertrophic cardiomyopathy (HCM), with a prevalence of up to 30% (33), representing a possible cause of ischemia and SCD (34) due to systolic lumen obliteration, but also persistent occlusion during diastole, as a result of impaired relaxation of the myocardium surrounding the anomalous segment. Although SCD has been ascribed to myocardial bridge in young people and in athletes, this feature is nowadays classified among the uncertain causes of SCD (7).

## Management of Asymptomatic Patients and Indication for Surgery

### Wrong Sinus Origin CAA

The incidental finding of a wrong sinus origin CAA *in vivo* is increasing due to the large use of non-invasive coronary artery imaging techniques or during angiography performed to detect atherosclerotic CA disease. This incidental diagnosis has a great impact on the management (medical treatment, interventional, or surgical repair) and the risk stratification of these patients. Most variants are benign. The best treatment for CAA is still debated, and a multidisciplinary approach is mandatory.

Moreover, after a CAA of wrong sinus origin is identified, clinical management should be based on nuclear stress coronary angiography to evaluate for the presence of atherosclerotic disease, and an intravascular ultrasound (IVUS) of the anomalous vessel. A grading of the CAA according to IVUS criteria has been proposed, by considering the amount of hypoplasia and the degree of lateral compression of the proximal vessel. Assessment of the fractional flow reserve (FFR) is also recommended together with IVUS, both at baseline and with dobutamine pharmacological stress. Cheezum et al. (35) published a useful comparison of all available anatomic tests used to characterize CAA. Moreover, while recognizing the potential values of ischemia provocative tests to assess the functional significance of CAA, a review of published data demonstrates that both exercise treadmill testing and stress myocardial perfusion imaging may yield false-positive and false-negative results. It is worthy to note that among the 27 young athletes who died suddenly with interarterial wrong sinus CAA reported by our group (11), six patients had a normal exercise treadmill test.

Guidelines (36, 37) recommend surgery (class I) in cases of CAA from the left or right sinus when it is associated with cardiac symptoms, or diagnostic evidence of stress-induced ischemia in the matching territory, or with high-risk coronary anatomy. Revascularization is also recommended for interarterial anomalous origin of the LCA, regardless of ischemia or symptoms. Despite these recommendations, the optimal management of patients with interarterial CAA is still debated. In a recent series of 66 middle-aged individuals with newly diagnosed CAA, mid-term outcome was not statistically different to a matched control cohort without CAA, regardless of whether CAA with or without interarterial course were present (38).

According to Cheezum et al. (35), in all cases of clinically suspected interarterial wrong sinus CAA, imaging with CT or MR is recommended to visualize anatomic features such as the proximal vessel obstruction that may guide surgical decision making. While in anomalous LCA with interarterial course surgical treatment should be always discussed, a conservative approach is reasonable in asymptomatic individuals with anomalous RCA with interarterial course, no proximal vessel narrowing, and no evidence of ischemia. The optimal management strategy likely varies as a function of individual age, presentation, anatomy, and physiology.

### Anomalous Origin of CA From the Pulmonary Artery

In this setting, surgical repair (mostly by reimplantation of the anomalous CA on the aortic root) is considered mandatory as soon as instrumental diagnosis is finalized (39). Concomitant repair of ischemic mitral regurgitation is usually not indicated unless anatomical abnormalities are associated. Even though left ventricular wall motion abnormalities, perfusion deficits, and myocardial scarring may remain in many patients, myocardial function improvement is expected in most cases within a few years after repair, if this was performed early in infancy (40).

### Myocardial Bridge

The major challenge is again the functional assessment for decision making when dealing with the incidental finding of myocardial bridge by angiography (“milking effect”) and/or CT (41).

Stress single-photon emission CT can detect reversible myocardial perfusion defects in those patients, with a correlation between the amount of ischemia and the degree of systolic luminal narrowing.

Coronary physiological measurements during pharmacological infusion are also helpful.

Imaging by IVUS can reveal the characteristic “half-moon” sign, an echolucent area between the bridged coronary segment and epicardial tissue that persists throughout the cardiac cycle. However, both in symptomatic patients and in those with an “incidental” finding by angiography or CT, there is no consensus whether further diagnostic studies of myocardial bridge are needed before therapy.

## Recommendations for Sport Activity in Athletes With CAA

Official consensus guidelines for eligibility/disqualification decisions in competitive athletes with CAA are available at international and at national levels (42, 43).

In the recent European Society of Cardiology (ESC) 2020 Guidelines on sports cardiology and exercise in patients with cardiovascular disease, evaluation with imaging tests to identify high-risk patterns and an exercise stress test to check for ischemia is recommended in individuals with either left or right wrong sinus CAA (class IIa, level C).

In asymptomatic individuals with wrong sinus CAA without interarterial course or a slit-like orifice with reduced lumen and/or intramural course, competition may be considered, after adequate counseling on the risks, provided there is absence of inducible ischemia (class IIb, level C). After surgical repair, sport participation may be considered 3 months after surgery, at the earliest, if they are asymptomatic and there is no evidence of inducible myocardial ischemia or complex cardiac arrhythmias during maximal exercise stress tests (class IIb, level C). Participation in most competitive sports with a moderate and high cardiovascular demand among individuals with wrong sinus CAA with an acutely angled take-off or an anomalous course between the large vessels is not recommended (class III, level C) (42).

These recommendations reflect what has been written in the 2017 update of the Italian COCIS guidelines for sport activity (43). Moreover, in this document the anomalous origin of the LCX from the right is eventually mentioned separately, recognizing the benign behavior in the absence of the signs and symptoms of ischemia.

As far as myocardial bridge is concerned, the ESC guidelines say that participation in competitive and leisure-time sports should be considered in asymptomatic individuals without inducible ischemia or ventricular arrhythmia during maximal exercise testing (Class IIa, level C). Competitive sports are not recommended in individuals with myocardial bridge and persistent ischemia or complex cardiac arrhythmias during maximal exercise stress testing (class III, level C). The clinical evaluation of individuals with myocardial bridge includes the morphologic assessment of the anatomical anomaly (i.e., depth and overall length of the tunneled vessel) and the presence of inducible ischemia. A positive inotropic and positive chronotropic stress test is the best approach to demonstrate myocardial ischemia.

Such recommendations again reflect those proposed in the 2017 update of the Italian COCIS guidelines for sport activity.

**Figure 3:**
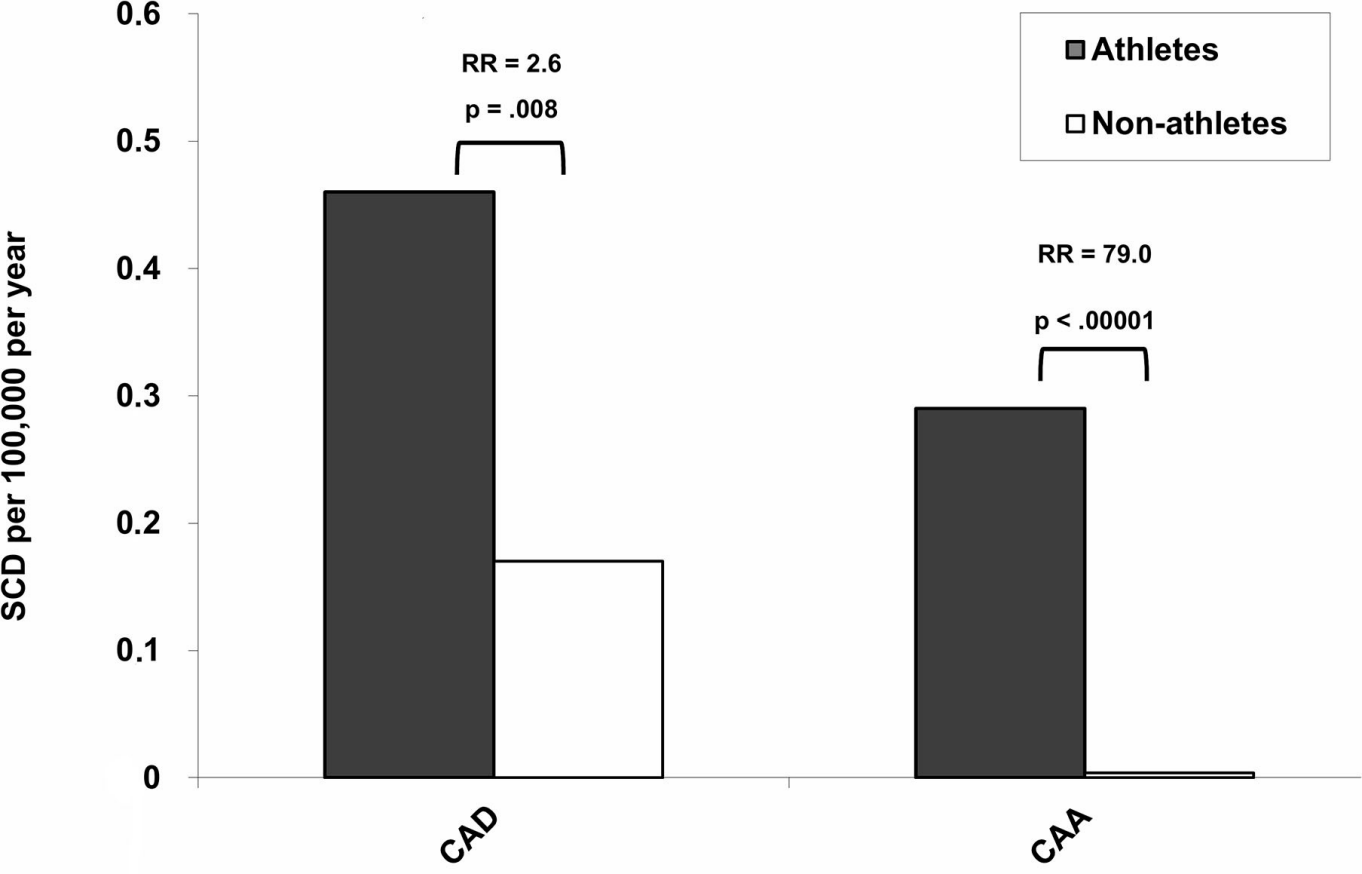
Incidence and relative risk (RR) for sudden cardiac death (SCD) for atherosclerotic coronary artery disease (CAD) and coronary artery anomalies (CAA) among athletes and non-athletes [modified from Corrado et al. (52)].

Although atherosclerotic CA disease is the major determinant of acute coronary syndrome and SCD, CAA represents a significant cause of SCD in the young and in athletes, particularly in the context of exercise (9, 11, 14, 15, 44–51). In the prospective cohort study of all young people of the Veneto Region of Italy, sports activity was associated with an increased risk of SCD. In particular, sport triggered SCD in those athletes who were affected by cardiovascular conditions predisposing to ventricular arrhythmias during effort. The higher risk of SCD in athletes was strongly related to underlying cardiovascular diseases such as CAA (RR 79, CI 10 to 3,564; *p* < 0.0001) (Figure 3), arrhythmogenic right ventricular cardiomyopathy (RR 5.4, CI 2.5 to 11.2; *p* < 0.0001), and premature atherosclerotic CA disease (RR 2.6, CI 1.2 to 5.1; *p* = 0.008) (52) (Figure 3). Table 1 lists autopsy-proven studies reporting the prevalence of CAA as the cause of SCD in the young and/or in athletes (9, 52, 55, 58, 60, 61, 63, 64, 68, 69).

**Table 1:** Prevalence of CAA in major (≥100 cases) autopsy series of sudden cardiac death in the young.

**Authors (Reference)**	**Year**	**Time**	**Location**	**Population**	**Age**	**N. SCD**	**Sex, *M* (%)**	**CAA, *N* (%)**
Burke et al. (9)	1991	1981–1988	Maryland, United States	Athletes Non athletes	14–40	34 656	31 (91) 501 (76)	4 (12) 8 (1.2))
Drory et al. (53)	1991	1976–1985	Israel	General	9–39	162	134 (82.7)	1 (0.6)
Corrado et al. (52)	2003	1979–1999	Veneto region, Italy	Athletes Non athletes	1–35	55 245	50 (90.9) 170 (69.3)	9 (16.3) 5 (2)
Van Camp et al. (54)	1995	1983–1993	US high schools and colleges	Athletes	13–22	100	92 (92)	16 (16)
Maron et al. (46)	1996	1985–1995	United States	Athletes	<35	134	120 (89.5)	31 (23.1)
Wisten et al. (55)	2002	1992–1999	Swedish	General	15–35	181	132 (72.9)	7 (3.9)
Morentin et al. (56)	2003	1991–1998	Bizkaia county, Spain	General	1–35	107	ND	ND
Doolan et al. (57)	2004	1994–2002	New South Wales, Sydney, Australia	General	<35	193	125 (64.7)	ND
Eckart et al. (58)	2004	1977–2001	Brooke Army Medical Center, San Antonio, Texas, United States	General	18–35	126	111 (88.1)	21 (16.7)
Puranik et al. (59)	2005	1995–2004	Eastern part of Sydney, Australia	General	5–35	241	189 (78.4)	5 (2.1)
Di Gioia et al. (60)	2006	2001–2005	Lazio region, Italy	General	1–35	100	69 (69)	4 (4)
Maron et al. (49)	2009	1980–2006	United States	Athletes	13–25	1049	937 (89.3)	119 (11.3)
Eckart et al. (62)	2011	1998–2008	Personnel from the Department of Defense, United States	General	18–35	298	282 (94.6)	12 (4.0)
Margey et al. (63)	2011	2005–2007	Ireland	General	15–35	116	90 (77.5)	2 (1.7)
Winkel et al. (64)	2011	2000–2006	Denmark	General	1–35	314	210 (67)	3 (0.9)
Pilmer et al. (65)	2013	2008	Ontario, Canada	General	2–40	174	133 (76.4)	ND
de Noronha et al. (66)	2014	2007–2009	United Kingdom	General	0–35	422	ND	5 (1.2)
Risgaard et al. (67)	2014	2007 - 2009	Denmark	General	12–49	439	317 (72.2)	4 (0.9)
Bagnall et al. (68)	2016	2010–2012	Australia and New Zealand	General	1–35	490	353 (72)	ND
Maron et al. (61)	2016	1980-2011	United States	Athletes	<35	842	747 (88.7)	158 (18.8)
Finocchiaro et al. (69)	2016	1994-2014	United Kingdom	Athletes	<35	258	ND	13 (5.0)

*SCD, sudden cardiac death; CAA, coronary artery anomaly; ND, not determinable*.

Because electrocardiograms, both 12-leads basal and stress test, have a scarce sensibility, the presence of alarming signs or symptoms particularly on effort should lead to perform non-invasive and invasive imaging tools for early identification of CAA and decision about sports eligibility.

## Author Contributions

All authors have participated in the research and/or article preparation and approved the final article.

## Conflict of Interest

The authors declare that the research was conducted in the absence of any commercial or financial relationships that could be construed as a potential conflict of interest.
